# P-1579. Demonstrating Measurable Impact Within Infection Prevention and Control Through Certification

**DOI:** 10.1093/ofid/ofaf695.1758

**Published:** 2026-01-11

**Authors:** Jessica Dangles, Mayar Al Mohajer

**Affiliations:** Certification Board of Infection Control and Epidemiology, Arlington, VA; Baylor College of Medicine, Houston, TX

## Abstract

**Background:**

The Certification Board of Infection Control and Epidemiology (CBIC) developed the Advanced Leadership Certification in Infection Prevention (AL-CIP) to recognize advanced expertise in infection prevention and healthcare epidemiology. Unlike traditional exams, AL-CIP uses a portfolio-based model, allowing candidates to demonstrate leadership through real-world, longitudinal achievements.

**Figure:**
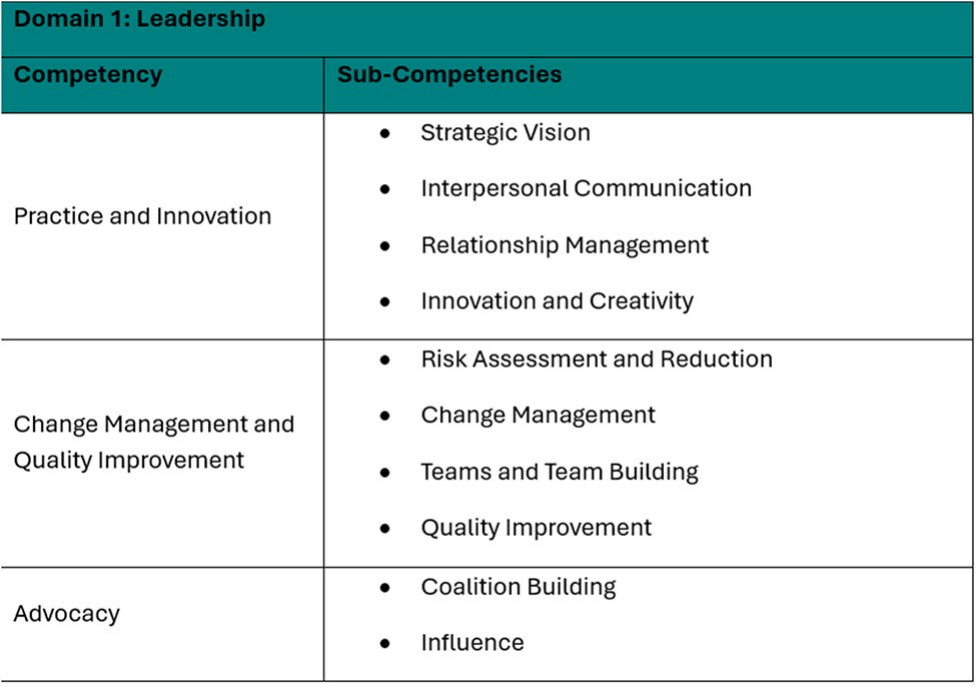
Competency Framework: Domain 1 - Leadership

**Figure:**
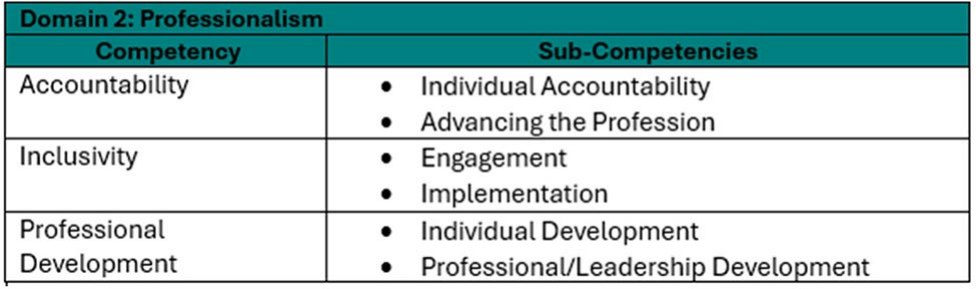
Competency Framework: Domain 2 - Professionalism

**Methods:**

A panel of ten infection prevention and control (IPC) subject matter experts (SMEs), supported by a psychometric consultant, conducted a job task analysis to identify key competencies for advanced IPC leadership. These findings informed the development of a portfolio-based certification framework.

To validate the assessment process, a beta-testing phase was conducted. Candidates submitted portfolios aligned with defined sub-competencies during a one-month window. Volunteer raters received training, completed calibration exercises, and independently evaluated portfolios using a standardized platform.

Data from the beta test were analyzed for rating consistency and alignment with provisional standards. A follow-up survey captured rater feedback, which informed final adjustments to content presentation, rationale requirements, and scoring criteria.

**Results:**

A total of 141 candidates from 10 countries submitted portfolios during the first testing window, with an average of 17 years’ experience in infection prevention and control (IPC). Submissions included both new applicants and re-applicants from the beta test.

Candidates addressed at least 50% of sub-competencies within two domains: Leadership and Professionalism, submitting eight pieces of evidence with rationale to demonstrate impact and alignment with the criteria.

Ongoing analysis includes content selection trends, rater reliability, and evaluation consistency, which will guide future refinements to the assessment process.

**Conclusion:**

The AL-CIP provides IPC professionals with a platform to demonstrate the measurable impact of their leadership. By showcasing strategic vision, change management, and program improvement, certified leaders help advance IPC practices and reduce infection risk across diverse settings.

**Disclosures:**

All Authors: No reported disclosures

